# Impact of optical coherence tomography on diagnostic decision‐making by UK community optometrists: a clinical vignette study

**DOI:** 10.1111/opo.12613

**Published:** 2019-04-17

**Authors:** Anish Jindal, Irene Ctori, Bruno Fidalgo, Priya Dabasia, Konstantinos Balaskas, John G Lawrenson

**Affiliations:** ^1^ Division of Optometry and Visual Science City University of London London UK; ^2^ Moorfields Eye Hospital NHS Foundation Trust London UK

**Keywords:** glaucoma, optic nerve, optical coherence tomography, optometrists, retina

## Abstract

**Purpose:**

In recent years, there has been widespread investment in imaging technologies by community optometrists in the UK, most notably optical coherence tomography (OCT). The aim of the current study was to determine the value of OCT in the diagnosis of posterior segment diseases in a representative sample of community optometrists using a clinical vignette methodology.

**Methods:**

A group of community optometrists (*n* = 50) initially completed a standardised training package on OCT interpretation followed by a computer‐based assessment featuring 52 clinical vignettes, containing images of healthy (*n* = 8) or glaucomatous (*n* = 18) discs or healthy (*n* = 8) or diseased (*n* = 18) fundi. Each vignette featured either a single fundus/disc photographic image, or a combination of a fundus/disc image with the corresponding OCT scan. An expert panel confirmed that the fundus images presented alone and those in combination with OCT data were of a similar level of difficulty and that the cases were typical of those seen in primary care. For each case, the optometrist selected their diagnosis from a pull‐down list and reported their confidence in their decision using a 10‐point Likert scale. Pairwise comparisons of the fundus image alone and fundus image/OCT combination were made for both diagnostic performance and confidence.

**Results:**

The mean percentage of correct diagnoses using fundus imaging alone was 62% (95% CI 59–64%) and for the combination of fundus image/OCT was 80% (95% CI 77–82%). The mean false negative rate with fundus alone was 27% reducing to 13% with the OCT combination. Median confidence scores for fundus imaging alone was 8.0 (IQR 7.0–8.0) and 8.3 (IQR 8.0–9.0) for the combination. Improvements in performance and confidence were statistically significant (*p* < 0.001).

**Conclusion:**

The results from this vignette study suggests that OCT improves optometrists’ diagnostic performance compared to fundus observation alone. These initial results suggest that OCT provides valuable additional data that could augment case‐finding for glaucoma and retinal disease; however, further research is needed to assess its diagnostic performance in a routine clinical practice setting.

## Introduction

As the major provider of primary eye care, UK optometrists play a key role in the opportunistic detection of both symptomatic and asymptomatic eye disease. They also initiate the vast majority of referrals into secondary care.^1^, ^2^, ^3^, ^4^, ^5^, ^6^, ^7^

Although optometrists referral accuracy seems to improve with clinical experience,^8^, ^9^ decision‐making in the diagnosis of glaucoma and retinal disease is often associated with considerable uncertainty. In practice, the health of the optic disc or macula is judged subjectively based on direct or indirect fundoscopy. High false positive and false negative rates have been reported in disc assessment by optometrists.^10^, ^11^ Similarly, a recent prospective study of optometrist referrals for neovascular AMD reported satisfactory performance in identifying symptoms, but poorer performance in recognising clinical signs.^7^

In the UK, and internationally, there has been a shift towards integrating advanced imaging technologies, particularly optical coherence tomography (OCT), into routine optometric practice.^12^, ^13^, ^14^, ^15^ However, it is not clear whether the incorporation of advanced imaging leads to an improved diagnostic performance. A recent study in Australia using case vignettes found that the diagnostic accuracy of macular disease is only marginally improved with the incorporation of advanced imaging techniques compared to colour fundus photography alone; the additional information from advanced imaging led to increased numbers of false positives and a greater tendency to refer cases to secondary care.^16^

The benefit of the widespread adoption of OCT on case‐finding for ocular disease by community optometrists in the UK is unclear. The primary aim of the current study is to determine the value of OCT in the diagnosis of posterior segment diseases in a representative sample of community optometrists using a clinical vignette methodology.

Clinical vignettes simulate realistic patient interactions and are widely used to measure variation in the diagnosis and management of disease across a range of medical specialities.^17^ Vignettes have been validated against unannounced standardised patients and case record abstraction as a measure of quality of care.^18^, ^19^ They offer a number of advantages, including control of case mix; the economies of scale means that they can be administered simultaneously to a large group of clinicians. For the current study, the cases were drawn from patients participating in a large cross sectional study of elderly subjects with a range of pathologies that would be typical of those seen in routine optometric practice.^20^

## Materials and methods

### Participating optometrists

UK registered community optometrists were recruited from several sources. An invitation to participate in the study was sent either directly by e‐mail, via posters distributed to local community practices, or through contacting Local Optical Committees. An incentive was offered, in terms of Continuing Education and Training (CET) accreditation for completing the online training.

Optometrists expressing an interest were asked to complete a survey to determine their eligibility for the study. The survey also asked for information on mode of practice (locum, independent, multiple group), postgraduate qualifications, and any further training or professional development undertaken. To be included in the study, participants had to be registered in the UK and needed to be practising in the community for at least 2 days per week. Optometrists were excluded if they had ever participated in any age‐related macular degeneration (AMD) shared care schemes, or previously worked in a medical retina or glaucoma secondary care clinics.

The study was approved by the School of Health Sciences Research Ethics Committee, City, University of London, and complied with the tenets of the Declaration of Helsinki. Written and informed consent was obtained from all participants prior to taking part in the study.

### Standardised online training

We anticipated that participating optometrists would vary in their experience of interpreting OCT data and decided *a priori* to develop a bespoke online training programme to familiarise participants with the principles of OCT interpretation, and specifically, with the characteristics of the data output from the iVue spectral domain OCT (SD‐OCT) (http://www.optovue.com). The training consisted of a 1 h online lecture (delivered via the university's virtual learning system) and links provided to relevant publications on OCT interpretation. The lecture covered the principles of OCT, interpretation of quantitative and qualitative data outputs and clinical examples of retinal and optic nerve pathology.

### Sourcing of clinical data

**Figure 1:**
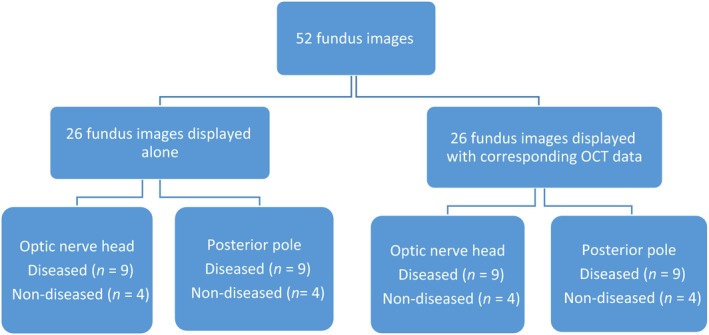
Flow diagram of the image allocation for the assessment.

Fundus and OCT images selected for the study were taken from a dataset, which was previously collected in a prospective community‐based cross‐sectional study.^20^ OCT scans of the disc and macula were captured using the IVue SD‐OCT (software version 3.2.0.42) and fundus photographs (45°) were taken through dilated pupils using the Topcon model TRC‐NW8F mydriatic/non‐mydriatic retinal camera (http://www.topconmedical.com). The patients were diagnosed at the time of data capture following a standard ophthalmic examination and using a validated reference standard; the definition regarding glaucoma classification have been described elsewhere. 20 Twenty‐six fundus images were chosen from the dataset, consisting of a mixture of normal eyes and eyes showing pathology; a similar set of 26 images with their corresponding OCT data files were also selected (Table S1). The image sets were of good quality and free from artefacts. Seventy per cent of the images in each set contained an ocular abnormality (*Figure* 1); this proportion was similar to the posterior segment abnormalities detected in the above cross‐sectional study. All clinical data used in the case vignettes was anonymised and patients had previously consented for their OCT scans and fundus images to be used for teaching and research purposes.

**Table 1:** Demographic characteristics of the participants (*n* = 50). Multiple refers to high street chains with practices throughout the UK

	Median (IQR)	*n*	%
A. No. years qualified	10 (4–19)		
B. Gender			
Female		31	62%
Male		19	38%
C. Setting of primary practice			
Independent		16	32%
Multiple		17	34%
Locum		17	34%
D. No. optometrists working in community primary care		50	100%
E. No. days working in community practice in a week	4 (3–5)		
F. No. optometrists working in secondary care		5	10%
G. No. days working in secondary care in a week	2 (1–2.5)		
H. Optometrists using fundus photography routinely		42	84%
I. Optometrists using OCT routinely		15	30%
J. Optometrists with postgraduate qualifications specific to glaucoma		8	16%
K. Optometrists with postgraduate qualifications specific to medical retina		1	2%
L. Previous attended training/courses regarding OCT			
OCT manufacturer		6	12%
Distance learning continued education and training		2	4%
Own practice/company		7	14%

### Expert panel

An independent expert panel was convened to (1) ensure that the fundus images to be presented alone, and those presented in combination with OCT data, were of a similar level of difficulty; and (2) to confirm that the cases were typical of those seen in primary care. The panel comprised five clinicians with expertise in medical retina and glaucoma (including two consultant ophthalmologists), an academic optometrist, an experienced community optometrist, and a hospital optometrist. The panel were asked to independently view both sets of fundus photographs and grade the level of difficulty of each image to diagnose the condition from the photograph alone using a 10‐point Likert scale. They were also asked to state whether the conditions were representative of a primary care case mix. All members of the panel agreed that the conditions were representative. Similarly, the level of difficulty scored by the expert panel was equivalent for the optic nerve and retinal disease cases between the two image sets.

### Case vignettes

**Figure 2:**
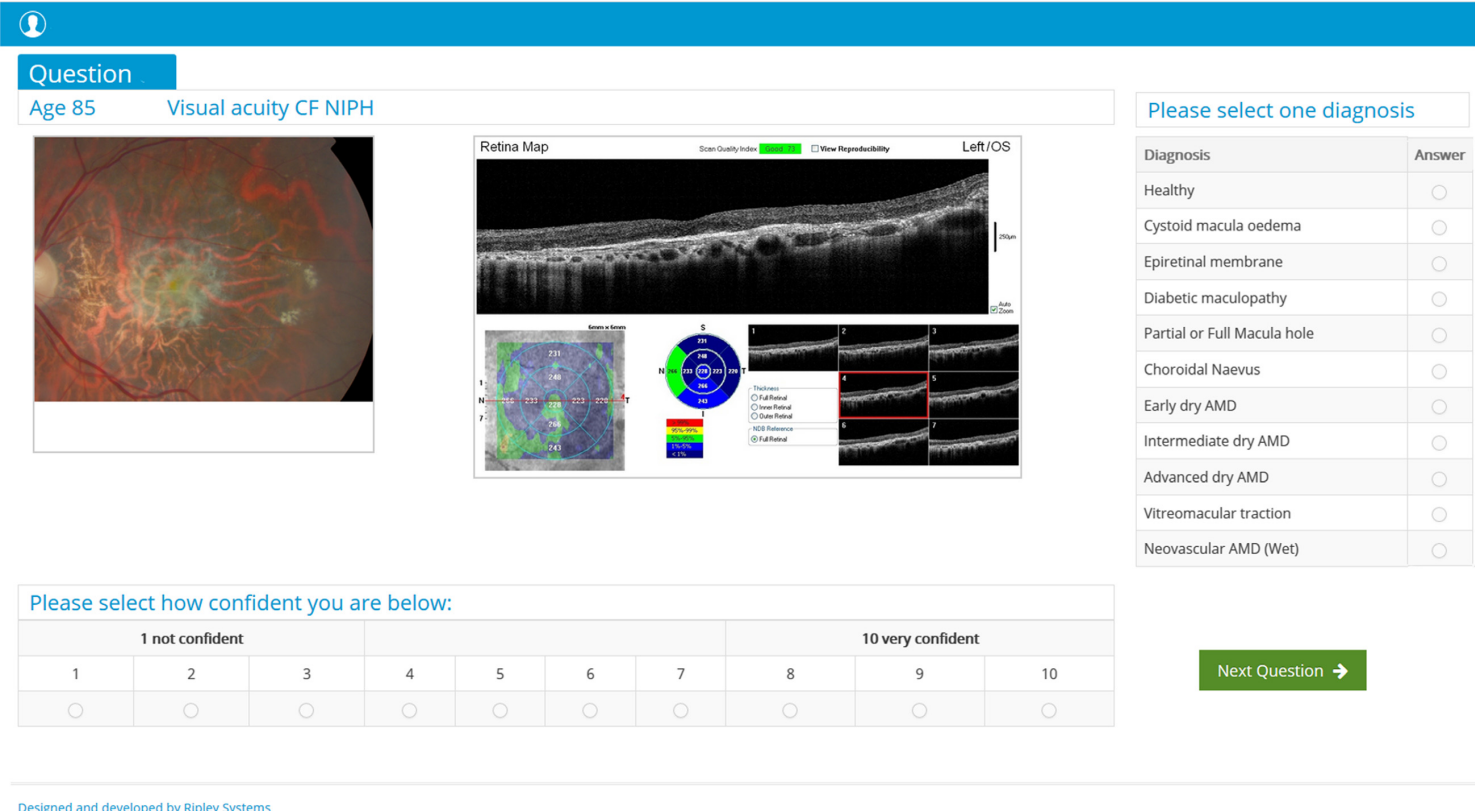
Question illustrating the central retina with the corresponding optical coherence tomography (OCT) retinal map. OCT data included seven B‐scans displaying sections of the macula from superior to inferior; macular thickness map that is colour coded according to the machines normative database.

**Figure 3:**
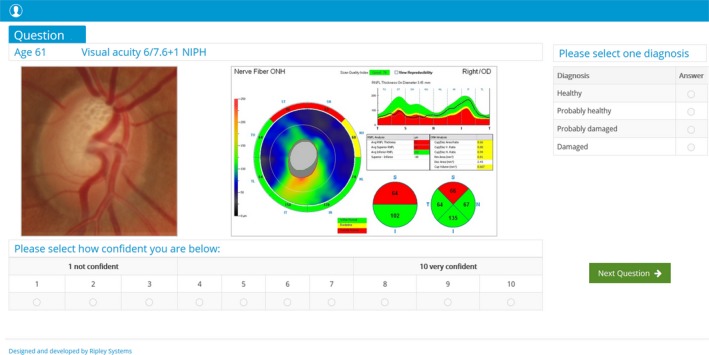
Question showing an optic disc with its accompanying optical coherence tomography (OCT) data. OCT data included retinal nerve fibre layer (RNFL) thickness with colour coded comparisons to the normative database in their respective quadrants; average overall RNFL thickness and hemifield thickness; and optic nerve head analysis displaying cup to disc ratios and volumetric analysis.

Case vignettes displayed a monoscopic fundus image, the age of the patient, the best‐corrected visual acuity and pinhole acuity gathered at time of original data capture. The fundus image showed either an optic disc or posterior pole of the retina; 26 of the vignettes were combined with a corresponding spectral‐domain OCT analytical report. For the posterior pole images, participants were asked to select a diagnosis from a pull‐down menu containing a list of 11 retinal conditions including a ‘healthy’ option (*Figure* 2). For the disc image, they were asked to classify the disc as either (1) Healthy, (2) Probably healthy, (3) Probably damaged, or (4) Damaged (*Figure* 3). Following each clinical decision, participants were asked to rate their confidence in their decision using a 10‐point Likert scale. The order of vignettes was randomised by a random number generator and presented using a specifically developed software package produced by Ripley Systems Ltd (http://www.ripleysystems.co.uk).

Prior to the start of the assessment, the vignettes were independently piloted for clarity, layout and content by three community optometrists. These data were not used in the final analysis.

### Assessment

Participants completed the assessment during a single 1 h session, held at the university. The vignettes were viewed under standardised viewing conditions on calibrated computer monitors. Before the assessment, participants were briefed as to the nature of the assessment and were shown demonstration vignettes. Participants were also briefed on the classification system used for the assessment for age‐related macular degeneration.^21^ In addition, participants were told that each vignette related either to a normal eye or an eye showing a single ophthalmic diagnosis. In addition, it was confirmed that there was no evidence of amblyopia and that the only risk factor provided was age.

### Sample size

A minimum of 51 images was needed to detect a statistically significant difference of 20% in performance; between the two diagnostic methods with 95% confidence and 80% power.^22^ For the sample size calculation, a median specificity of 74% was assumed based on previous studies involving community optometrists in detecting glaucomatous discs using disc images.^23^, ^24^, ^25^ For the number of participants making a clinical decision, a formal sample size calculation was not performed. However, we aimed to recruit a suitable number of community‐based optometrists who are representative of those working in the UK. From a previous study of glaucoma decision‐making using a similar computer based assessment, a sample of 53 optometrists provided a sufficiently narrow confidence interval for diagnostic performance in a disc assessment task.^24^

### Statistical analysis

Statistical analysis was performed using SPSS Statistics software version 24.0 (http://www.ibm.com/SPSS_statistics). To calculate the overall diagnostic performance for each participant, responses for each case vignette were converted into a binary score (one mark for a correct diagnosis and zero for an incorrect diagnosis). In the case of incorrect responses, the false positive and false negative rates was determined. A false negative was defined as a case showing an ocular disease incorrectly diagnosed as healthy; a false positive was a normal case incorrectly diagnosed as diseased. In cases where the correct diagnosis was either glaucoma suspect or glaucoma, a correct mark was given in both cases if the user answered either damaged or probably damaged, similarly if the disc was healthy, ‘probably healthy’ would be an acceptable answer. For AMD, a mark was given if the participant classified the disease as either intermediate or early. For all other retinal conditions, only the correct diagnosis was acceptable and alternative diagnoses were deemed to be incorrect.

Parametric and non‐parametric paired *t*‐tests were used to compare diagnostic performance and Wilcoxon tests for the confidence scores between fundus and OCT combination data sets. The Mann‐Whitney test was used for subgroup analyses based on participant gender, practice setting or years of experience post‐qualification. Linear regression was used to compare practitioner confidence against years qualified. For all tests, *p* < 0.05 was considered statistically significant.

## Results

### Participant characteristics

Sixty‐two participants completed the eligibility questionnaire and 12 participants were excluded: three participants worked <2 days a week in community practice and nine optometrists had previously worked in a medical retina or glaucoma secondary care clinic. Fifty optometrists were included in the final analysis and completed all case vignettes.

Characteristics of participants are summarised in *Table* 1. Participants had a median of 10 years post‐registration experience with the majority female (62%). Approximately equal numbers were either working as locums or based in independent or multiple practices. Less than one‐fifth of participants had additional qualifications related to either glaucoma or medical retina. Although the majority (84%) used fundus photography routinely, less than one‐third used OCT in their practice or had undertaken previous training in OCT interpretation.

**Table 2:** Confidence scores of participants (Median (IQR)); *p* values were calculated using the Wilcoxon sign rank test

	Fundus confidence	Combination confidence	*p*
Total confidence median (IQR)	8.0 (7.0–8.0)	8.3 (8.0–9.0)	<0.001
Disc confidence median (IQR)	8.0 (7.0–8.0)	9.0 (8.0–9.0)	<0.001
Retina confidence median (IQR)	8.0 (7.0–8.0)	9.0 (8.0–9.0)	<0.001

### Overall diagnostic performance

**Figure 4:**
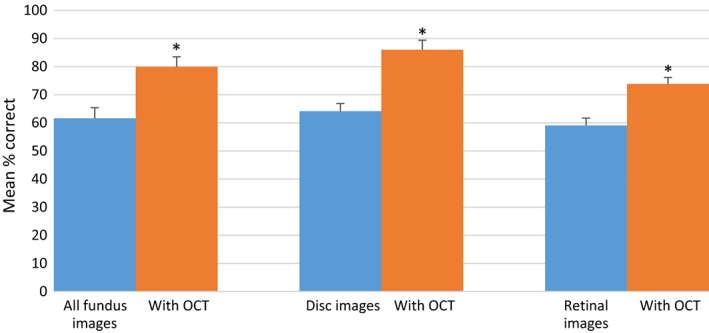
Correctly identified mean percentage score of total, optic discs and retinal cases using fundus alone and combination optical coherence tomography (OCT). Error bars show 95% confidence intervals. * indicates a statistically significant difference (*p* < 0.001).

**Figure 5:**
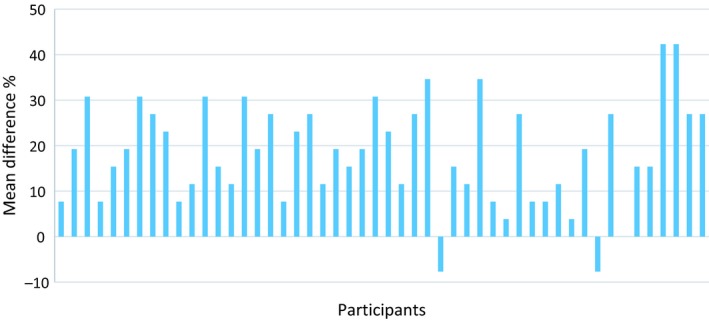
Difference scores between performance using the optical coherence tomography OCT combination and fundus image alone. Positive scores indicate an improvement with the combination.

The mean percentage of case vignettes correctly identified using fundus imaging alone was 62% (16/26 cases) (95% CI 59–64%), increasing to 80% (21/26 cases) (95% CI 77–82%) for the fundus image/OCT combination. Pairwise comparisons between these two sets of images were statistically significant (paired *t*‐test, *t*_49_ = 11.40, *p* < 0.001), statistically significant improvements were also seen for the OCT combination for both disc (paired *t*‐test, *t*_49_ = 11.33, *p* < 0.001) and retinal cases (paired *t*‐test, *t*_49_ = 6.35, *p* < 0.001) (*Figure* 4). Nearly all of the optometrists performed better with the supplementary OCT clinical data (94%), one participant (2%) showed no improvement, and two participants (4%) performed worse (*Figure* 5).

### False negative and false positive rates

**Figure 6:**
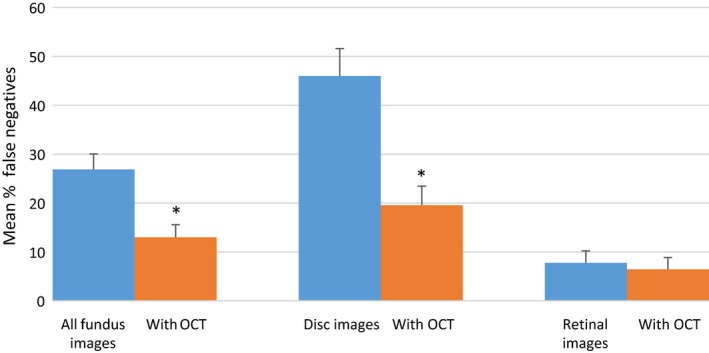
False negative rates. Error bars show 95% confidence intervals. * indicates a statistically significant difference (*p* < 0.05).

The overall false negative rate was 27% for cases consisting of a fundus image alone. This reduced to 13% for the fundus image/OCT combination. Although this difference was statistically significant (paired *t*‐test, *t*_49_ = 8.30, *p* < 0.001), a significant reduction was seen only for the disc scenarios (*Figure* 6).

**Figure 7:**
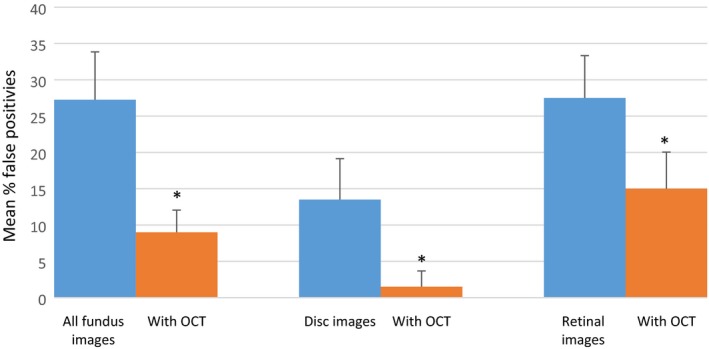
False positive rates. Error bars show 95% confidence intervals. * indicates a statistically significant difference (*p* ≤ 0.002).

The mean false positive rate using fundus image alone and for the combination with OCT were 27% and 9% respectively, which was statistically significant (paired *t*‐test, *t*_49_ = 6.33, *p* < 0.001). Significant reductions were observed for both discs and retinal cases, (paired *t*‐test, *t*_49_ = 4.62, *p* < 0.001) and (paired *t*‐test, *t*_49_ = 3.29, *p* = 0.002) respectively (*Figure* 7).

### Subgroup analysis of performance

There was no difference in diagnostic performance based on participant gender, practice setting or years of experience post‐qualification. Although prior OCT training did not have an impact on overall performance, there was statistically significance improvement for retinal conditions with OCT (Mann‐Whitney test, *U* = 159.5, *p* = 0.026)) but not for discs. The overall performance of optometrists with additional qualifications in either glaucoma or medical retina with the OCT combination was better than those without (Mann‐Whitney test, *U* = 96, *p* = 0.024).

### Confidence

The median confidence of diagnostic decisions was high (median 8 out of 10), and confidence only marginally improved with the addition of the OCT data for both retinal and disc cases (*Table* 2).

There was no statistically significant difference in diagnostic confidence between genders or practice setting. Similarly, further qualifications or experience in using the OCT did not influence confidence. However, there was a positive correlation between number of years qualified and confidence in decisions for all vignettes consisting of the fundus image alone, (Pearson correlation, *r* = 0.324, *p* = 0.022). However, this was not the case for the OCT combination.

## Discussion

There is increasing recognition that case‐finding for age‐related eye disease can be augmented by the application of advanced imaging technologies, which can aid the diagnosis of glaucoma^20^, ^26^ and the identification of retinal disease.^16^ OCT in particular, is rapidly gaining popularity in primary eye care. Successive surveys in the UK showed that the use of OCT in optometric practice increased seven fold within a decade.^13^, ^27^ This number is expected to rise substantially following the announcement that the largest UK‐based optometry chain will be incorporating OCTs in all of their practices within the next few years.^28^

### Main study findings

In this novel study, the performance of community optometrists was assessed in diagnosing a range of pathologies affecting the posterior segment using case vignettes containing fundus images alone, compared to vignettes with fundus images and the corresponding OCT data. With the OCT combination, the overall percentage of cases that were correctly classified increased by an average of approximately 20%. A statistically significant improvement was seen for the evaluation of glaucomatous optic neuropathy and retinal conditions. Over 90% of participants showed an improvement in their overall diagnostic accuracy when fundus images were combined with OCT, with performance from some individuals increasing by over 40%. Furthermore, with the additional information provided by OCT, the proportion of false negatives and false positives were significantly reduced. Although the overall diagnostic performance of optometrists with additional qualifications in either glaucoma or medical retina was superior with the OCT combination, performance was unaffected by the number of years of post‐registration experience. Participant confidence in their diagnostic decisions was high for both image sets, with minimal improvement in confidence with the OCT combination. The study was insufficiently powered to determine differences in confidence scores for false positives and false negative diagnoses.

### Disc assessment

Several authors have previously investigated the performance of community optometrists in the subjective assessment of optic discs.^24^, ^25^, ^29^ The disc images and OCT scans used in the present study were taken from participants recruited into a community‐based, cross‐sectional study to assess the performance of technologies for glaucoma case‐finding.^20^ Within each image 70% of discs were from patients with confirmed or suspect primary open angle glaucoma (presence of glaucomatous optic neuropathy with or without a corresponding visual field defect) and 30% were normal. The overall diagnostic accuracy based on a 2D observation of disc images alone was approximately 65%. However, the false negative rate was high (46%). These results were similar to the findings of a study of Australian optometrists using 2D disc observations.^29^ Since the original diagnoses of glaucomatous optic neuropathy was based on dilated indirect ophthalmoscopy, it is possible that diagnostic accuracy may have improved with 3D visualisation. A UK study, using stereoscopic photographs of healthy and glaucomatous discs, reported an overall accuracy of 80%.^25^ Although it could be argued that in standard clinical practice, a diagnosis of glaucoma is usually based on a combination of disc observation and visual field assessment, optic nerve damage is often the first clinically detectable sign of the disease. For example, in randomised controlled trials of patients with ocular hypertension, 40–60% of cases converting to glaucoma showed optic disc changes before reproducible visual field damage.^30^, ^31^ The current study provides evidence that disc evaluation can be augmented by additional information on the integrity of the retinal nerve fibre layer provided by OCT. We found a significant improvement in overall diagnostic accuracy with the OCT combination and a reduction in false positive and false negative rates.

### Diagnosis of retinal disease

A major advantage in using OCT to diagnose retinal and macular diseases is its ability to provide high‐resolution, cross‐sectional images of the retina and perform quantitative segmental analysis of retinal layers.^32^ The Retinal Disease Screening Study compared fundus photography with OCT imaging in 158 asymptomatic subjects and concluded that OCT was more sensitive than fundus photography for the detection of retinal irregularities and was able to detect significantly more clinically relevant disease.^33^ The present study showed an improvement in performance for the diagnosis of retinal conditions with the addition of data from OCT and a corresponding reduction in the false positive rate. The false negative rate was low (<10%) for both sets of vignettes. To our knowledge, this is the first study to look at the impact of OCT on optometrists’ diagnostic decisions for a range of retinal and macular diseases. A recently published vignette study from Australia evaluated the effect of advanced imaging (including OCT) on optometrists’ decision‐making for the diagnosis and management of AMD.^16^ In this study, the use of fundus photography alone resulted in an accurate diagnosis of AMD in 61% of cases. Overall diagnostic accuracy improved by a modest 5% with advanced imaging and the additional information led to an increased false positive rate and a greater tendency to refer to secondary care. The authors concluded that a lack of training in interpreting the results of advanced imaging might explain the findings. Although participants in our study had varying experience of OCT interpretation upon recruitment, we attempted to mitigate this via standardised online training prior to carrying out the assessment.

### Participant variables affecting performance

Our study showed that the overall diagnostic performance was not influenced by the demographic characteristics of participants, the number of years of clinical experience nor previous exposure to OCT. However, the 18% of participants with additional postgraduate qualifications performed statistically better than those without when interpreting the OCT combination. This was consistent with the findings of Hadwin and colleagues,^25^ who reported that optometrists with higher qualifications had a higher overall accuracy in stereoscopic optic disc assessment. Significantly, previous studies have shown that short‐term didactic training has little impact on clinical decision‐making in glaucoma^24^, ^29^ and it would appear that a combination of ongoing training with regular feedback on clinical decisions is more likely to improve performance.^34^, ^35^

### Strengths and limitations of the study

One of the strengths of the study was the relevance of the case‐mix. The images used in the vignettes were taken from a cohort of patients who presented in primary care. The two sets of vignettes were externally validated to ensure a similar level of difficulty and were pilot tested. In addition, all participants received a standardised online training programme in OCT interpretation, which was similar to training given to new OCT users.

In terms of demographics and mode of practice, the study participants were broadly representative of community optometrists working in the UK.^36^, ^37^ However, we may have recruited optometrists who were more confident in their diagnostic skills, which could explain the high confidence scores with both image sets.

Since the aim of the study was to investigate the ability of optometrists to recognise disc damage and features on retinal images, the vignettes did not provide all of the information that would normally be available in primary care such as presence of risk factors, data from visual field plots, IOP readings, inter‐ocular differences and the results a stereoscopic fundus examination. Furthermore, the inclusion of unequivocally ‘normal’ vignettes may have unintentionally improved the diagnostic performance of the OCT. It has previously been demonstrated that effectiveness of the OCT in detecting glaucoma significantly decreases when evaluated against a more clinically relevant control group, with suspicious‐looking discs compared to a control group with no suspicious findings.^38^ The results may therefore not be fully representative of the participants’ diagnostic performance in a ‘real world’ setting.

## Conclusions

There has been widespread investment in imaging technologies by community optometrists in the UK, most notably OCT. The results from this study suggests that OCT improves optometrists’ diagnostic performance and confidence. These initial results suggest that OCT provides valuable additional data that could augment case‐finding for glaucoma and retinal disease. Whilst the improvement in diagnostic performance is encouraging, OCT should still be employed judiciously in a routine clinical practice setting. It is also important that clinicians are appropriately trained in OCT data interpretation and appreciate the limitations as well as the strengths of the technology.

## Disclosure

The authors report no conflicts of interest and have no proprietary interest in any of the materials mentioned in this article.

## Supporting information


**Table S1.** Case mix of the conditions shown in the clinical vignettes.

## References

[1] Bowling B , Chen S & Salmon J . Outcomes of referrals by community optometrists to a hospital glaucoma service. Br J Ophthalmol 2005; 89: 1102–1104. 16113358 10.1136/bjo.2004.064378 PMC1772809

[2] Kelly SP , Wallwork I , Haider D & Qureshi K . Teleophthalmology with optical coherence tomography imaging in community optometry. Evaluation of a quality improvement for macular patients. Clin Ophthalmol 2011; 5: 1673–1678. 22174576 10.2147/OPTH.S26753 PMC3236713

[3] Azuara‐Blanco A , Burr J , Thomas R , Maclennan G & McPherson S . The accuracy of accredited glaucoma optometrists in the diagnosis and treatment recommendation for glaucoma. Br J Ophthalmol 2007; 91: 1639–1643. 17537783 10.1136/bjo.2007.119628 PMC2095552

[4] Bell R & O'Brien C . The diagnostic outcome of new glaucoma referrals. Ophthalmic Physiol Opt 1997; 17: 3–6. 9135805

[5] O'Connor PM , Harper CA , Brunton CL *et al* Shared care for chronic eye diseases: perspectives of ophthalmologists, optometrists and patients. Med J Aust 2012; 196: 646–650. 22676881 10.5694/mja11.10856

[6] Davey CJ , Green C & Elliott DB . Assessment of referrals to the hospital eye service by optometrists and GPs in Bradford and Airedale. Ophthalmic Physiol Opt 2011; 31: 23–28. 21070302 10.1111/j.1475-1313.2010.00797.x

[7] Muen WJ & Hewick SA . Quality of optometry referrals to neovascular age‐related macular degeneration clinic: a prospective study. JRSM Short Rep U6 2011; 2: 64. 10.1258/shorts.2011.011042 PMC3166265 21912730

[8] Parkins DJ , Benwell MJ , Edgar DF & Evans BJW . The relationship between unwarranted variation in optometric referrals and time since qualification. Ophthalmic Physiol Opt 2018; 38: 550–561. 30175473 10.1111/opo.12580

[9] Davey CJ , Scally AJ , Green C , Mitchell ES & Elliott DB . Factors influencing accuracy of referral and the likelihood of false positive referral by optometrists in Bradford, United Kingdom. J Optom 2016; 9: 158–165. 26614021 10.1016/j.optom.2015.10.007 PMC4911451

[10] Ratnarajan G , Kean J , French K , Parker M & Bourne R . The false negative rate and the role for virtual review in a nationally evaluated glaucoma referral refinement scheme. Ophthalmic Physiol Opt 2015; 35: 577–581. 26088949 10.1111/opo.12224

[11] Keenan J , Shahid H , Bourne RR , White AJ & Martin KR . Cambridge community Optometry Glaucoma Scheme. Clin Exp Ophthalmol 2015; 43: 221–227. 25070417 10.1111/ceo.12398

[12] Jamous KF , Kalloniatis M , Hayen A *et al* Application of clinical techniques relevant for glaucoma assessment by optometrists: concordance with guidelines. Ophthalmic Physiol Opt 2014; 34: 580–591. 25103462 10.1111/opo.12146

[13] Dabasia PL , Edgar DF , Garway‐Heath DF & Lawrenson JG . A survey of current and anticipated use of standard and specialist equipment by UK optometrists. Ophthalmic Physiol Opt 2014; 34: 592–613. 25160893 10.1111/opo.12150

[14] Ly A , Nivison‐Smith L , Zangerl B , Assaad N & Kalloniatis M . Self‐reported optometric practise patterns in age‐related macular degeneration: optometric practise patterns in AMD. Clin Exp Optom 2017; 100: 718–728. 28266060 10.1111/cxo.12528

[15] Kiely PM , Cappuccio S & McIntyre E . Optometry Australia Scope of Practice Survey 2015. Clin Exp Optom 2017; 100: 260–269. 28295595 10.1111/cxo.12538

[16] Ly A , Nivison‐Smith L , Zangerl B , Assaad N & Kalloniatis M . Advanced imaging for the diagnosis of age‐related macular degeneration: a case vignettes study: imaging age‐related macular degeneration. Clin Exp Optom 2018; 101: 243–254. 28994139 10.1111/cxo.12607 PMC5873408

[17] Veloski J , Tai S , Evans AS & Nash DB . Clinical vignette‐based surveys: a tool for assessing physician practice variation. Am J Med Qual 2005; 20: 151–157. 15951521 10.1177/1062860605274520

[18] Peabody JW , Luck J , Glassman P , Dresselhaus TR & Lee M . Comparison of vignettes, standardized patients, and chart abstraction: a prospective validation study of 3 methods for measuring quality. JAMA 2000; 283: 1715–1722. 10755498 10.1001/jama.283.13.1715

[19] Peabody JW , Luck J , Glassman P *et al* Measuring the quality of physician practice by using clinical vignettes: a prospective validation study. Ann Intern Med 2004; 141: 771–780. 15545677 10.7326/0003-4819-141-10-200411160-00008

[20] Dabasia PL , Fidalgo BR , Edgar DF , Garway‐Heath DF & Lawrenson JG . Diagnostic accuracy of technologies for glaucoma case‐finding in a community setting. Ophthalmology 2015; 122: 2407–2415. 26411836 10.1016/j.ophtha.2015.08.019

[21] Ferris FL , Wilkinson CP , Bird A *et al* Clinical classification of age‐related macular degeneration. Ophthalmology 2013; 120: 844–851. 23332590 10.1016/j.ophtha.2012.10.036 PMC11551519

[22] Hajian‐Tilaki K . Sample size estimation in diagnostic test studies of biomedical informatics. J Biomed Inform 2014; 48: 193–204. 24582925 10.1016/j.jbi.2014.02.013

[23] Ly A , Nivison‐Smith L , Hennessy MP & Kalloniatis M . Collaborative care of non‐urgent macular disease: a study of inter‐optometric referrals. Ophthalmic Physiol Opt 2016; 36: 632–642. 27790767 10.1111/opo.12322 PMC5129555

[24] Myint J , Edgar DF , Murdoch IE & Lawrenson JG . The impact of postgraduate training on UK optometrists’ clinical decision‐making in glaucoma. Ophthalmic Physiol Opt 2014; 34: 376–384. 24754430 10.1111/opo.12126

[25] Hadwin SE , Redmond T , Garway‐Heath DF *et al* Assessment of optic disc photographs for glaucoma by UK optometrists: the Moorfields Optic Disc Assessment Study (MODAS). Ophthalmic Physiol Opt 2013; 33: 618–624. 23634792 10.1111/opo.12066

[26] Azuara‐Blanco A , Banister K , Boachie C *et al* Automated imaging technologies for the diagnosis of glaucoma: a comparative diagnostic study for the evaluation of the diagnostic accuracy, performance as triage tests and cost‐effectiveness (GATE study). Health Technol Assess 2016; 20: 1–168. 10.3310/hta20080 PMC4781562 26822760

[27] Myint J , Edgar DF , Kotecha A , Murdoch IE & Lawrenson JG . A national survey of diagnostic tests reported by UK community optometrists for the detection of chronic open angle glaucoma. Ophthalmic Physiol Opt 2011; 31: 353–359. 21535068 10.1111/j.1475-1313.2011.00844.x

[28] Association of Optometrists . OCT rollout in every Specsavers announced. *Optometry Today* https://www.aop.org.uk/ot/industry/high-street/2017/05/22/oct-rollout-in-every-specsavers-announced (Accessed 30/11/18).

[29] Yoshioka N , Wong E , Kalloniatis M *et al* Influence of education and diagnostic modes on glaucoma assessment by optometrists. Ophthalmic Physiol Opt 2015; 35: 682–698. 26432198 10.1111/opo.12247

[30] Keltner JL , Johnson CA , Anderson DR *et al* The association between glaucomatous visual fields and optic features in the ocular treatment study nerve head hypertension. Ophthalmology 2006; 113: 1603–1612. 16949445 10.1016/j.ophtha.2006.05.061

[31] Miglior S . Predictive factors for open‐angle glaucoma among patients with ocular hypertension in the European Glaucoma Prevention Study. Ophthalmology 2007; 114: 3–9. 17070596 10.1016/j.ophtha.2006.05.075

[32] Health Quality Ontario . Optical coherence tomography for age‐related macular degeneration and diabetic macular edema: an evidence‐based analysis. Ont Health Technol Assess Ser 2009; 9: 1–22. PMC3377511 23074517

[33] Ouyang Y , Heussen FM , Keane PA , Sadda SR & Walsh AC . The retinal disease screening study: retrospective comparison of nonmydriatic fundus photography and three‐dimensional optical coherence tomography for detection of retinal irregularities. Invest Ophthalmol Vis Sci 2013; 54: 5694–5700. 23847317 10.1167/iovs.13-12043

[34] Theodossiades J , Murdoch I & Cousens S . Glaucoma case finding: a cluster‐randomised intervention trial. Eye 2004; 18: 483–490. 15131679 10.1038/sj.eye.6700676

[35] Patel UDM , Murdoch IE & Theodossiades J . Glaucoma detection in the community: does ongoing training of optometrists have a lasting effect? Eye 2006; 20: 591–594. 16021189 10.1038/sj.eye.6702000

[36] College of Optometrists . The Optical Workforce Survey Full report. https://www.college-optometrists.org/the-college/research/research-projects/optical-workforce-survey2.html (Accessed 30/11/18).

[37] General Optical Council: Equality and Diversity Monitoring Report 2016‐2017. http://www.optical.org/filemanager/root/site_assets/edi/2016-17_goc_edi_monitoring_report.pdf (Accessed 30/11/18).

[38] Rao HL , Kumbar T , Addepalli UK *et al* Effect of spectrum bias on the diagnostic accuracy of spectral‐domain optical coherence tomography in glaucoma. Invest Ophthalmol Vis Sci 2012; 53: 1058–1065. 22266520 10.1167/iovs.11-8463

