# Supplementary material for: Impact of optical coherence tomography on diagnostic decision‐making by UK community optometrists: a clinical vignette study
Source: Ophthalmic Physiol Opt. 2019 Apr 17;39(3):205–15. doi: 10.1111/opo.12613 (PMC6849707; doi:10.1111/opo.12613)
Supplement: Supplementary file 1 — Table S1. Case mix of the conditions shown in the clinical vignettes. [file OPO-39-205-s001.docx]

| **Fundus alone (n=26)** | | | **OCT combination (n=26)** | |
| --- | --- | --- | --- | --- |
| Median age (IQR) | 66 (65-72) | | 66 (65-74) | |
| Ethnicity No. (%)  Caucasian  African origin  South Asian  Asian | 24 (92%)  1 (4%)  0 (0%)  1 (4%) | | 17 (65%)  5 (19%)  3 (12%)  1 (4%) | |
| Diagnosis | | No. | Diagnosis | No. |
| Healthy disc | | 4 | Healthy disc | 4 |
| Glaucoma suspect | | 5 | Glaucoma suspect | 4 |
| Glaucoma | | 4 | Glaucoma | 5 |
| Healthy retina | | 4 | Healthy retina | 4 |
| Vitreo-macular traction | | 1 | Vitreo-macular traction | 1 |
| Epiretinal membrane | | 2 | Epiretinal membrane | 2 |
| Diabetic maculopathy | | 1 | Diabetic maculopathy | 2 |
| Choroidal naevus | | 1 | Choroidal naevus | 1 |
| Advanced dry AMD | | 1 | Advanced dry AMD | 1 |
| Macular hole | | 1 | Macular hole | 1 |
| Early AMD | | 1 | Early AMD | 0 |
| Intermediate AMD | | 1 | Intermediate AMD | 1 |

Supplementary table 1. Case mix of the conditions shown in the clinical vignettes.
